# Supplementary material for: RAGCell: retrieval-augmented generation as supervision for versatile single-cell analysis
Source: Bioinformatics. 2026 Oct 1;42(10):btag732. doi: 10.1093/bioinformatics/btag732 (PMC13675104; doi:10.1093/bioinformatics/btag732)
Supplement: Supplementary file 1 [file btag732_supplementary_data.zip › RAGCell_Supplementary.pdf]

Supplementary Material

RAGCell: Retrieval-Augmented Generation as Supervision for Versatile Single-cell Analysis

Tianyu Liu et al.

A Introduction to Incorporated Datasets

CITE-seq and ASAP-seq PBMC dataset. The CITE-seq and ASAP-seq PBMC datasets contain multimodal data from both control and stimulated conditions. After preprocessing, 4,644 CITE-seq cells and 4,502 ASAP-seq cells associated with 17,441 genes are included in the experiments. The CITE-seq data includes seven categories: B cells, Effector CD4+ T cells, Effector CD8+ T cells, Monocytes cells, NK cells, Naive CD4+ T cells, and Naive CD8+ T cells. The ASAP-seq data includes nine categories: B cells, DC cells, Effector CD4+ T cells, Effector CD8+ T cells, Monocytes cells, NK cells, Naive CD4+ T cells, Naive CD8+ T cells, and others. This dataset can be downloaded from GSE156478 (`https://www.ncbi.nlm.nih.gov/geo/query/acc.cgi?acc=GSE156478`).

Liver dataset. We create a Liver dataset with two batches from two different studies. We manually unify the cell type labels of two datasets by regular expressions to remove the mismatched spelling. We use the same Scanpy pre-processing step to normalize gene expression, unify gene names and create two batches including MacParland and Aizarani for training/testing. We have considered both directions.

PBMC 10K dataset. The PBMC 10K dataset includes two batches of scRNA-seq data from human PBMCs of a healthy donor. After preprocessing, this dataset features 3,346 differentially expressed genes. The first batch and the second batch comprise 7,982 cells and 4,008 cells, respectively. Cell groups were labeled by Seurat Satija *et al.* (2015) and categorized into nine types: B cells, CD4+ T cells, CD8+ T cells, CD14+ monocytes, dendritic cells, NK cells, FCGR3A+ monocytes, megakaryocytes, and others. This dataset can be downloaded from the scVI tools Virshup *et al.* (2023); Gayoso *et al.* (2022) (`https://scvi-tools.org/`) using the API scvi.data.pbmc_dataset.

BMMC dataset. The BMMC dataset uses the CITE-seq protocol and comprises paired measurements of scRNA-seq and protein abundance in BMMCs. This dataset includes cells from 12 healthy human donors, organized into 12 batches. After preprocessing, the data encompass 90,261 cells and each cell contains 13,953 genes and 134 surface proteins. There are 45 distinct cell types in this dataset. This dataset can be downloaded from GSE194122 (`https://www.ncbi.nlm.nih.gov/geo/query/acc.cgi?acc=GSE194122`).

GSE149383-Erlotinib. The GSE149383 dataset comprises 2,739 human lung cancer cells and 8,380 associated genes, along with their respective sensitivity properties to the drug Erlotinib. This dataset can be downloaded from GSE149383 (`https://www.ncbi.nlm.nih.gov/geo/query/acc.cgi?acc=GSE149383`).

GSE117872-Cisplatin. The GSE117872 dataset comprises 1,302 human oral squamous cancer cells and 22,744 associated genes, along with their respective sensitivity properties to the drug Cisplatin. This dataset can be downloaded from GSE117872 (`https://www.ncbi.nlm.nih.gov/geo/query/acc.cgi?acc=GSE117872`).

B Introduction to Compared Methods

To evaluate the performance of `RAGCell` for downstream tasks, we benchmarked our method against a range of state-of-the-art (SOTA) approaches. For tasks like cell type annotation, batch effect correction, and multi-omics data integration, we compared `RAGCell` with four scFMs, including scBERT, scGPT, GenePT, and scELMo. For the rare cell type identification task, we employed scCAD as the framework and replaced the original cell embeddings with cell representations obtained by our approach. For the drug sensitivity prediction task, we compared `RAGCell` with both specialist models like vanilla Transformer, scDEAL, and generalist models like GPT-3.5.

scBERT Yang *et al.* (2022). scBERT is a pretrain-based scFM that utilizes a Performer cell encoder and employs masked gene modeling for pretraining on one million cells from the Panglao dataset Franzén *et al.* (2019). We used the pretrained model provided by the authors from (`https://drive.weixin.qq.com/s?k=AJEAIQdfAAoUxhXE7r#`) and validated downstream tasks according to their publicly available code from (`https://github.com/TencentAILabHealthcare/scBERT`).

All other settings were kept consistent with those provided by the authors.

scGPT Cui *et al.* (2024). scGPT is another pretrain-based scFM that employs a decoder-based architecture combined with attention mask for autoregressive pretraining on 33 million cells. The authors provided several pretrained model weights, with pretraining data ranging from 1.8 million heart cells to 33 million normal human cells. We utilized the model pretrained on the largest dataset from (https://drive.google.com/drive/folders/1oWh_-ZRdhtoGQ2Fw24HP41FgLoomVo-y) and conducted downstream task experiments following the authors' publicly available code from (https://github.com/bowang-lab/scGPT). All other settings were kept consistent with those provided by the authors.

Geneformer Theodoris *et al.* (2023). Geneformer is a pretrain-based scFM that uses a transformer encoder architecture for context-aware gene representation learning from rank-value encoded single-cell transcriptomes. The model was pretrained on approximately 30 million human single-cell transcriptomes from the Genecorpus-30M dataset, enabling transfer to diverse downstream tasks such as cell type annotation, perturbation prediction, and gene network analysis. The authors provided pretrained model weights through Hugging Face, and we utilized the publicly available pretrained Geneformer model from (https://huggingface.co/ctheodoris/Geneformer) for downstream task experiments.

scFoundation Hao *et al.* (2024). scFoundation is a pretrain-based scFM that employs a transformer-based architecture to learn universal cell representations from large-scale single-cell gene expression profiles. The model was pretrained on over 50 million human single-cell transcriptomes, enabling broad transferability across downstream tasks such as cell type annotation, perturbation response prediction, and gene expression reconstruction. The authors provided pretrained model weights, and we utilized the publicly available pretrained scFoundation model from (https://github.com/biomap-research/scFoundation) for downstream task experiments.

UCE Rosen *et al.* (2023). UCE is a pretrain-based scFM that employs a large transformer-based architecture to generate universal cell embeddings for single-cell transcriptomic data. The model was pretrained in a self-supervised manner on more than 36 million cells from over 300 datasets across human and seven other species, aiming to learn a unified biological latent space across tissues, datasets, and species. A key feature of UCE is that it tokenizes genes using protein-language-model-derived embeddings, enabling cross-species and even new-species generalization. The authors provided pretrained model weights and released the implementation publicly, and we utilized the pretrained UCE model from (https://github.com/snap-stanford/UCE) for downstream task experiments.

Cell2Sentence Levine *et al.* (2023). Cell2Sentence is a pretrain-based scFM that reframes single-cell transcriptomes as natural-language-like sentences where genes are ordered according to their expression ranks and processed using large language models. Instead of directly modeling continuous gene expression matrices, Cell2Sentence converts each cell into a sequence of gene tokens, enabling decoder-based language models to learn gene-gene context and generate biologically meaningful cell representations. The authors provided pretrained C2S models and tutorials for rank transformation, reconstruction, and downstream analysis, and we utilized the publicly available implementation from (https://github.com/vandijklab/cell2sentence) for downstream task experiments.

GenePT Chen and Zou (2023). GenePT is an LLM-based scFM that obtains text embeddings of features (such as genes and proteins) from LLM using text descriptions from the NCBI database as prompts. It then directly uses these LLM embeddings to construct cell embeddings. We employed the GenePT-w method, as provided by the authors from (https://github.com/yiqunchen/GenePT), to construct cell embeddings and validate them for downstream tasks. All other settings were kept consistent with those provided by the authors.

scELMo Liu *et al.* (2023). scELMo is another LLM-based scFM, which can be considered as an improved version of GenePT. It obtains embeddings through enhanced prompts to construct high-quality cell embeddings. We utilized the code provided by the authors from (https://github.com/HelloWorldLTY/scELMo) to build cell embeddings and validate them for downstream tasks. All other settings were kept consistent with those provided by the authors.

scCAD Xu *et al.* (2024). scCAD is a SOTA algorithm for rare cell type identification. It employs the PCA method for clustering to obtain cell embeddings. We conducted experiments using the publicly available code from (https://github.com/xuyp-csu/scCAD) and replaced the original cell embeddings with representations from our framework. Experimental analysis was performed using the cell embeddings generated by our approach. All other parameters of the algorithm were kept consistent with the original implementation.

Transformer Vaswani (2017). Transformer is a famous architecture and was initially designed for natural language processing tasks. It introduces self-attention mechanisms and provides standards for many other fields, including vision, audio, and scientific research. For the drug sensitivity prediction task,

all settings and parameters were kept consistent with the standard implementation.

scDEAL Chen *et al.* (2022). scDEAL is a specialist model designed for the drug sensitivity prediction task. It employs a neural network and tries to establish connections between genes and drug responses. The source code and implementation can be found at (`https://github.com/OSU-BMBL/scDEAL`). All settings and parameters were kept consistent with the original implementation.

LangCell Zhao *et al.* (2024). LangCell is a cell-text pretrained model that couples a Geneformer-style cell encoder with a text encoder and aligns the two through contrastive learning over cell-type descriptions, so that a cell can be annotated by matching it to type descriptions rather than to a fixed label set. Of the published methods it is the closest in design to RAGCell, since both introduce textual descriptions of cell identity into representation learning, and it is therefore the most informative comparison for isolating what the retrieval component contributes (`https://github.com/PharMolix/LangCell`).

GPT-3.5 and GPT-4o mini. GPT-3.5 is a reliable LLM developed by OpenAI. Based on the GPT-3 architecture, GPT-3.5 can understand and generate human-liked text, which could be employed for many tasks and applications, including the drug sensitivity prediction task. The use of GPT-3.5 API can be found at (`https://platform.openai.com/docs/models/gpt-3.5-turbo`). We also test the usage of GPT-4o mini in our ablation studies. API: `https://platform.openai.com/docs/models/gpt-4o-mini`

C Implementation Details

`RAGCell` employs the patch-based Transformer model as the cell model. Each cell is split into 128 patches for data tokenization. For cell-level knowledge databases, we employ the GPT-4o mini model to generate function descriptions and use the 'text-embedding-3-large' model to extract embeddings, with an embedding dimension of 3072. For feature-level knowledge databases, we leverage the GPT-3.5 model to generate function descriptions and use the 'text-embedding-ada-002' model to extract embeddings, with an embedding dimension of 1536. To map cell and text representations into a shared space, we use two MLPs as projection layers, reducing both representations to a low-dimensional space of 128 dimensions. All experiments are conducted using Pytorch with 80GB A100 GPUs as support. For cell-text alignment, we set the batch size to 256 and trained the cell model with the AdamW optimizer for 100 epochs. For finetuning tasks, we randomly split the datasets into 80% for training, 10% for validation, and 10% for testing. For zero-shot tasks, we ensure that there is no data leakage or overlap during the pretraining phase. The learning rate is set to 1.5e-4 during the pretraining stage and 1e-4 during the finetuning stage, respectively.

D Evaluation Metrics

Cell Type Annotation. For the cell type annotation task, we evaluated the model's performance using four common metrics for classification tasks: Accuracy, Precision, Recall, and F1 score. The calculation processes for each metric are as follows:

$$\text{Accuracy} = \frac{\text{TP} + \text{TN}}{\text{TP} + \text{TN} + \text{FP} + \text{FN}}, \tag{1}$$

$$\text{Precision} = \frac{1}{N}\sum_{i=1}^{N}\frac{\text{TP}_i}{\text{TP}_i + \text{FP}_i}, \tag{2}$$

$$\text{Recall} = \frac{1}{N}\sum_{i=1}^{N}\frac{\text{TP}_i}{\text{TP}_i + \text{FN}_i}, \tag{3}$$

$$\text{F1 Score} = \frac{1}{N}\sum_{i=1}^{N}\frac{2 \times \text{Precision}_i \times \text{Recall}_i}{\text{Precision}_i + \text{Recall}_i}, \tag{4}$$

where TP, TN, FP, and FN are short for true positives, true negatives, false positives, and false negatives, respectively. N denotes the number of samples per cell type.

Batch Effect Correction. For the batch effect correction task, we evaluated the model's performance using several common cell clustering metrics, specifically normalized mutual information (NMI), adjusted rand index (ARI), and average silhouette width (ASW). NMI can be calculated as:

$$\text{I}(U,V) = \sum_{i=1}^{|U|}\sum_{j=1}^{|V|} P(i,j)\log\left(\frac{P(i,j)}{P(i)P(j)}\right), \tag{5}$$

$$\text{NMI}(U,V) = \frac{I(U,V)}{\text{mean}(\text{H}(U),\text{H}(V))}, \tag{6}$$

where $P(i,j)$ is the probability that the i-th cluster in clustering U and the j-th cluster in clustering V occur simultaneously. $P(i)$ and $P(j)$ are the probabilities of the i-th cluster in U and the j-th cluster in V, respectively.

ARI can be calculated as:

$$\text{RI} = \frac{\text{TP} + \text{TN}}{\text{TP} + \text{TN} + \text{FP} + \text{FN}}, \tag{7}$$

$$\text{ARI} = \frac{\text{RI} - E[\text{RI}]}{\max(\text{RI}) - E[\text{RI}]}, \tag{8}$$

where TP, TN, FP, and FN are short for true positives, true negatives, false positives, and false negatives, respectively. $E[RI]$ is the expected RI of random labeling.

The calculation process for ASW can be represented as follows:

$$a(i) = \frac{1}{|C_i| - 1}\sum_{j \in C_i, j \neq i} d(i,j), \tag{9}$$

$$b(i) = \min_{k \neq i} \frac{1}{|C_k|} \sum_{j \in C_k} d(i, j), \tag{10}$$

$$s(i) = \frac{b(i) - a(i)}{\max\{a(i), b(i)\}}, \tag{11}$$

$$\text{ASW} = \frac{1}{N} \sum_{i=1}^{N} s(i), \tag{12}$$

where C_i and C_k are clusters containing sample points i and k, and $d(i, j)$ is the distance between sample points i and j. N denotes the number of cell samples.

Multi-omics Data Integration. For the multi-omics data integration task, we employed three metrics, namely NMI, ARI, and ASW, to evaluate the performance of the models. The calculation processes for these metrics have been previously described.

Rare Cell Type Identification. For the rare cell type identification task, we employed three metrics, namely Precision, Recall, and F1 score, to evaluate the performance of the models. The calculation processes for these metrics have been previously described.

Drug Sensitivity Prediction. For the drug sensitivity prediction task, we employed four metrics, namely Precision, Recall, F1 score, and Accuracy, to evaluate the performance of the models. The calculation processes for these metrics have been previously described.

E Additional Experiments

E.1 Ablation Study

To verify the effectiveness of incorporating priors from LLMs, we also provide some model variants by gradually removing text descriptions at each level, with results shown in Fig. S1. As illustrated in the figure, 'w/o feature-level' signifies that only cell-level descriptions were included, whereas 'w/o feature&cell-level' indicates the absence of all textual information. The results indicate performance declines among various model variants, which suggests the need of both cell-level and feature-level text descriptions in enhancing cell representations.

E.2 Additional Cell Type Annotation Results

We also present the cell type annotation confusion matrix results in Fig. S2, which highlights the superior performance of `RAGCell` compared to other scFMs. `RAGCell` achieves significantly higher annotation accuracy consistently across diverse cell types.

F Examples of Text Descriptions

Here we provide some examples of cell-level and feature-level text descriptions on different datasets in Fig. S3, Fig. S4, Fig. S5, Fig. S6, Fig. S7, and Fig. S8.

F.1 Additional experiments.

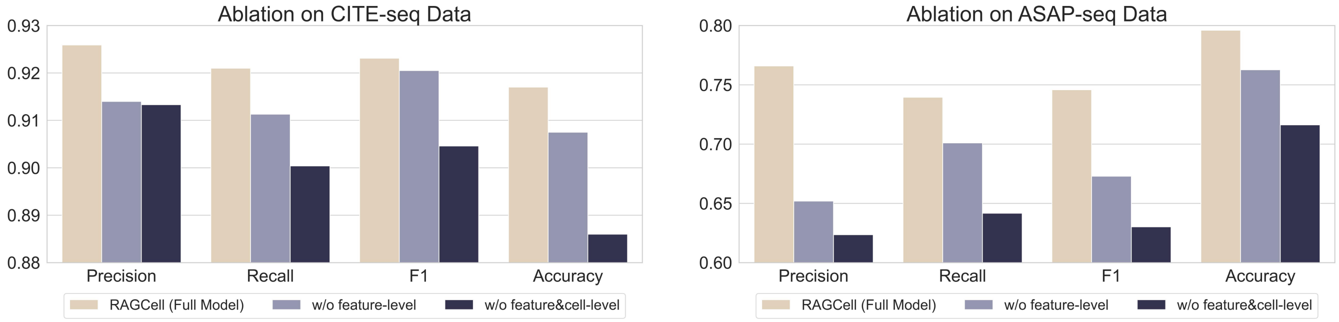

Figure S1: Ablation study on CITE-seq and ASAP-seq data.

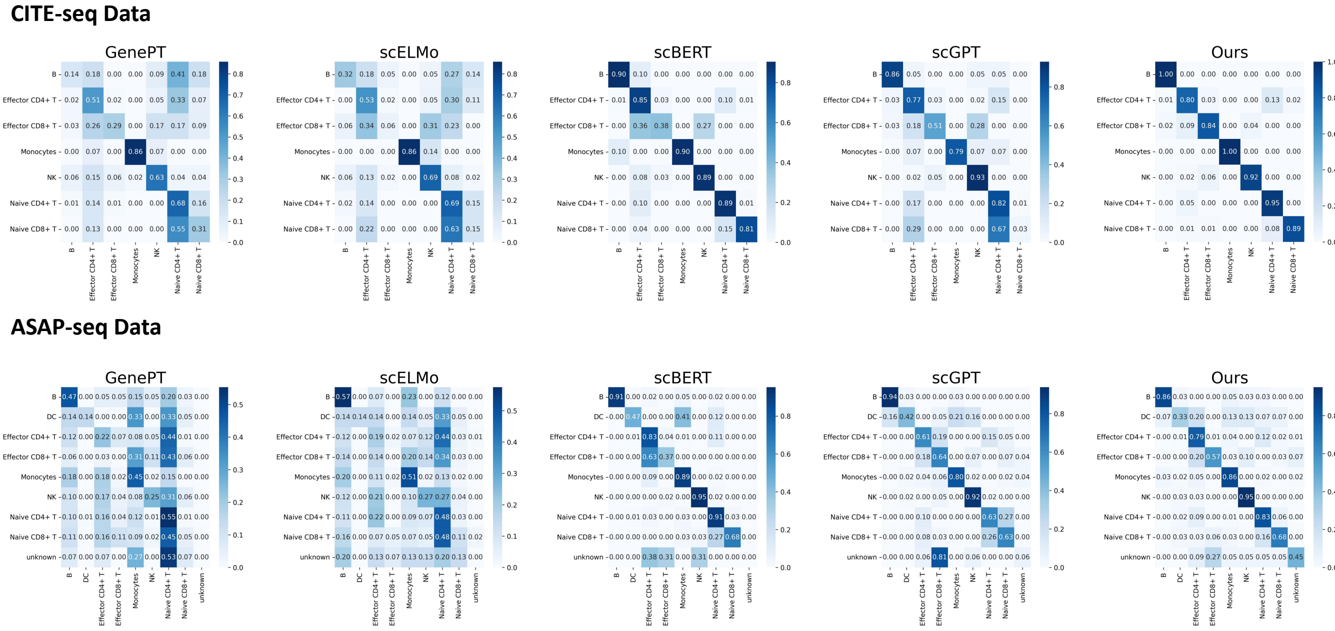

Figure S2: Confusion matrix results on CITE-seq and ASAP-seq data.

CITE-seq Data

Cell-level

Human: This is a single cell CITE sequence sample with a cell type of B. Please summarize its major function. Use academic language in one paragraph and think step by step.

LLM: B cells, a critical component of the adaptive immune system, primarily function to mediate humoral immunity through the production of antibodies. Derived from hematopoietic stem cells in the bone marrow, these lymphocytes undergo a complex maturation process, during which they develop the ability to recognize and bind specific antigens presented by pathogens. Upon activation by antigenic stimulation, often facilitated by T helper cells, B cells differentiate into plasma cells, which are responsible for secreting large quantities of antibodies. These antibodies play an essential role in neutralizing toxins, opsonizing pathogens for enhanced phagocytosis, and activating the complement system, thereby orchestrating an effective immune response. Additionally, B cells contribute to immune memory, enabling a more rapid and robust response upon subsequent exposures to the same antigen through the generation of memory B cells. Overall, the primary function of B cells is to provide protection against infections and to maintain immunological memory.

Feature-level

Human: Please summarize the major function of gene: CDK11B. Use academic language in one paragraph and and think step by step.

LLM: The CDK11B gene encodes a member of the cyclin-dependent kinase (CDK) family, which is integral to the regulation of the cell cycle, particularly in the G2 and M phases. Specifically, CDK11B functions as a serine/threonine kinase that associates with cyclins to phosphorylate target proteins, thereby facilitating various cellular processes including transcription regulation, cell proliferation, and differentiation. Notably, CDK11B also plays a crucial role in mRNA processing and spliceosome assembly, underscoring its involvement not only in cell cycle progression but also in the post-transcriptional modification of RNA. Dysregulation of CDK11B activity has been implicated in various cancers, highlighting its potential as a therapeutic target.

Figure S3: Examples of cell-level and feature-level (using gene as an example) text descriptions on CITE-seq data.

ASAP-seq Data

Cell-level

Human: This is a single cell ASAP sequence sample with a cell type of DC. Please summarize its major function. Use academic language in one paragraph and think step by step.

LLM: Dendritic cells (DCs) are pivotal antigen-presenting cells within the immune system, characterized primarily by their ability to capture, process, and present antigens to naïve T lymphocytes, thereby initiating and modulating adaptive immune responses. These cells reside in peripheral tissues and serve as sentinels, detecting pathogens and environmental changes. Upon encountering antigens, DCs undergo a maturation process that enhances their capacity to migrate to lymphoid tissues, where they express co-stimulatory molecules and major histocompatibility complex (MHC) molecules crucial for T cell activation. Additionally, DCs can secrete a variety of cytokines and chemokines, which not only fortify T cell responses but also influence the differentiation of other immune cells, such as B cells and natural killer cells. Importantly, the interaction between DCs and T cells is critical for establishing immune tolerance and preventing autoimmune reactions, highlighting the dual role of DCs in both instigating protective immunity and maintaining homeostasis within the immune landscape.

Feature-level

Human: Please summarize the major function of gene: SCNN1D. Use academic language in one paragraph and and think step by step.

LLM: The gene SCNN1D encodes the delta subunit of the epithelial sodium channel (ENaC), which plays a pivotal role in regulating sodium and fluid homeostasis in various epithelial tissues, particularly in the kidney and respiratory system. The major function of SCNN1D involves its contribution to the assembly and functional activity of the ENaC complex, which is essential for sodium absorption across epithelial cell membranes. This process is critical in maintaining extracellular fluid volume, blood pressure, and the overall electrolyte balance within the body. Dysregulation or mutations in SCNN1D can lead to pathophysiological conditions, such as pseudohypoaldosteronism type 1, characterized by impaired sodium reabsorption, hyperkalemia, and metabolic acidosis. Thus, SCNN1D is fundamentally important for cardiovascular and renal physiology, as well as overall homeostatic mechanisms.

Figure S4: Examples of cell-level and feature-level (using gene as an example) text descriptions on ASAP-seq data.

PBMC 10K Data

Cell-level

Human: This is a single cell RNA sequence sample with a cell type of Megakaryocytes. Please summarize its major function. Use academic language in one paragraph and think step by step.

LLM: Megakaryocytes are large, polyploid bone marrow-derived cells primarily responsible for the production of platelets, which are critical components of the hemostatic system. They originate from hematopoietic stem cells and undergo a process of endomitosis, resulting in an increase in nuclear DNA content without cell division, thus enhancing their functional capacity. The primary function of megakaryocytes is to synthesize and release platelets into the bloodstream, which is essential for the initiation and regulation of blood clotting, thereby preventing excessive bleeding during vascular injury. In addition to their role in hemostasis, megakaryocytes and platelets also participate in immune responses, inflammation, and wound healing, showcasing their multifaceted biological importance within the hematopoietic system. Furthermore, understanding megakaryocyte biology is crucial for the development of therapeutic strategies targeting platelet-related disorders and other hematological conditions.

Feature-level

Human: Please summarize the major function of gene: ISG15. Use academic language in one paragraph and think step by step.

LLM: ISG15 (Interferon-Stimulated Gene 15) is an important ubiquitin-like protein that plays a crucial role in the innate immune response, primarily acting as a mediator of antiviral activity. Upon stimulation by interferons, ISG15 is highly induced and subsequently conjugated to target proteins through a process known as ISGylation, which modulates their function and stability. This post-translational modification not only enhances the immune response against viral infections but also regulates various cellular processes, including protein degradation, gene expression, and inflammation. Furthermore, ISG15 has been implicated in the modulation of pathways leading to apoptosis and cell signaling, thereby influencing the overall cellular stress response. Collectively, the multifaceted functions of ISG15 position it as a critical player in the host defense mechanisms against pathogens and in the maintenance of cellular homeostasis.

Figure S5: Examples of cell-level and feature-level text descriptions on PBMC 10K data.

BMMC Data

Cell-level

Human: This is a single cell sample with a cell type of B1 B IGKC+. Please summarize its major function. Use academic language in one paragraph and think step by step.

LLM: The B1 B IGKC+ cell type represents a subset of B lymphocytes characterized by the expression of immunoglobulin kappa chain (IGKC) and is primarily involved in the production of antibodies, particularly in the context of innate-like immune responses. These cells are known for their ability to rapidly produce immunoglobulin M (IgM) antibodies in response to polysaccharide antigens, which are often found on the surface of pathogens. This rapid antibody production is crucial for the early defense against infections, particularly in mucosal tissues. Furthermore, B1 B cells play a significant role in maintaining immune homeostasis and contribute to the regulation of immune responses through the secretion of cytokines and the modulation of other immune cell types. Their unique developmental origin and functional properties distinguish them from conventional B2 cells, highlighting their importance in both innate and adaptive immunity.

Feature-level

Human: Please summarize the major function of gene: FAM41C. Use academic language in one paragraph and think step by step.

LLM: FAM41C (Family with Sequence Similarity 41 Member C) is a gene that encodes a protein implicated in various cellular processes, notably in the regulation of protein homeostasis and cellular stress responses. Its expression is noted to influence autophagy, a critical mechanism for maintaining cellular integrity through the degradation and recycling of damaged organelles and proteins. Additionally, FAM41C has been associated with the modulation of signaling pathways related to cell proliferation and apoptosis, suggesting a role in maintaining cellular health and viability. Recent research has also indicated its potential involvement in pathological conditions, including various cancers, where dysregulation of FAM41C expression may contribute to tumorigenesis and cancer progression. Overall, FAM41C serves as a pivotal factor in cellular maintenance and responses to stress, with implications for both normal physiological processes and disease states.

Figure S6: Examples of cell-level and feature-level text descriptions on BMMC data.

GSE149383_Erlotinib

Cell-level

Human: This is a single cell RNA sequence sample and its sensitivity to the drug Erlotinib is resistant. Please summarize its major function. Use academic language in one paragraph and think step by step.

LLM: The single-cell RNA sequencing analysis of the sample reveals a distinct transcriptional profile that underlies its resistance to the drug Erlotinib, an epidermal growth factor receptor (EGFR) inhibitor. This resistance may be attributed to several cellular mechanisms, including the aberrant activation of alternative signaling pathways, upregulation of efflux transporters, or mutations in the EGFR or downstream mediators that negate the therapeutic effects of Erlotinib. Furthermore, the expression of immune checkpoint molecules and inflammatory cytokines could indicate an adaptive immune response that counteracts the drug's efficacy. The identified gene expression patterns suggest that this cell type may play a critical role in tumor microenvironment dynamics, contributing to therapeutic failure through cell-autonomous survival strategies and modulation of local inflammation, thereby presenting significant implications for the development of alternative therapeutic strategies targeting resistance mechanisms in cancer treatment.

Figure S7: Examples of cell-level and feature-level text descriptions on GSE149383 data.

GSE117872_Cisplatin

Cell-level

Human: This is a single cell RNA sequence sample and its sensitivity to the drug Cisplatin is sensitive. Please summarize its major function. Use academic language in one paragraph and think step by step.

LLM: Cisplatin is a chemotherapeutic agent predominantly utilized in the treatment of various solid tumors, and its effectiveness hinges upon its ability to induce DNA damage, thereby triggering cellular apoptosis in cancerous cells. In the context of a single-cell RNA sequencing sample characterized by sensitivity to Cisplatin, several key functional pathways may be implicated. These include the activation of DNA repair mechanisms, regulation of apoptosis, and cellular stress response pathways. A sensitive response typically suggests an upregulation of intrinsic pro-apoptotic signals and downregulation of survival pathways, facilitating the eradication of malignant cells. Furthermore, gene expression profiling may reveal alterations in cell cycle regulation and metabolic pathways that enhance vulnerability to Cisplatin-induced cytotoxicity, thereby underscoring the complex interplay between pharmacological intervention and the molecular landscape of the tumor microenvironment. Overall, the major function of this sensitive sample can be framed within the context of promoting programmed cell death in response to genotoxic stress, thereby elucidating potential therapeutic targets for optimizing cancer treatment outcomes.

Feature-level

Human: Please summarize the major function of gene: DENND6B. Use academic language in one paragraph and think step by step.

LLM: DENND6B (DENN domain-containing 6B) is a gene that encodes a protein characterized by the presence of a DENN domain, which is implicated in the regulation of small GTPases, particularly those within the Rab family that are critical for vesicular trafficking processes. The major function of DENND6B involves facilitating the activation and subsequent signaling pathways of specific Rab GTPases, which are essential for the transport of vesicles within cells. This process plays a pivotal role in mediating intracellular communication and the regulated secretion of molecules, influencing cellular response mechanisms to various stimuli. Furthermore, DENND6B has been associated with various physiological processes, including neuronal development and immune system function, highlighting its potential significance in health and disease states. Overall, the regulation of Rab GTPases by DENND6B underscores its fundamental role in maintaining cellular homeostasis and proper cellular functionality.

Figure S8: Examples of cell-level and feature-level text descriptions on GSE117872 data.

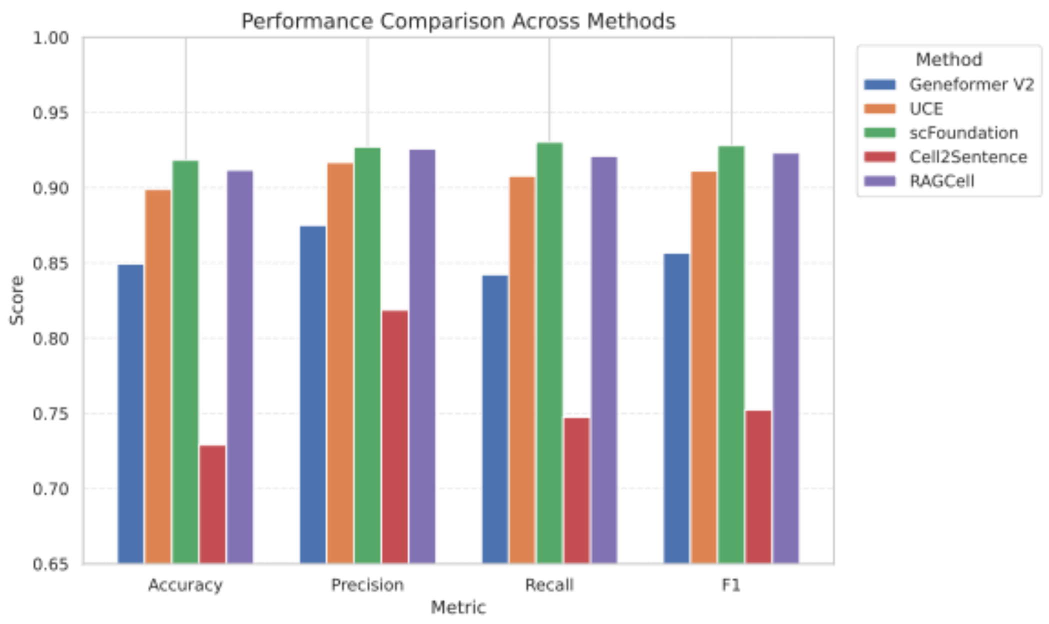

Figure S9: Benchmarking results with more methods for cell-type annotation.

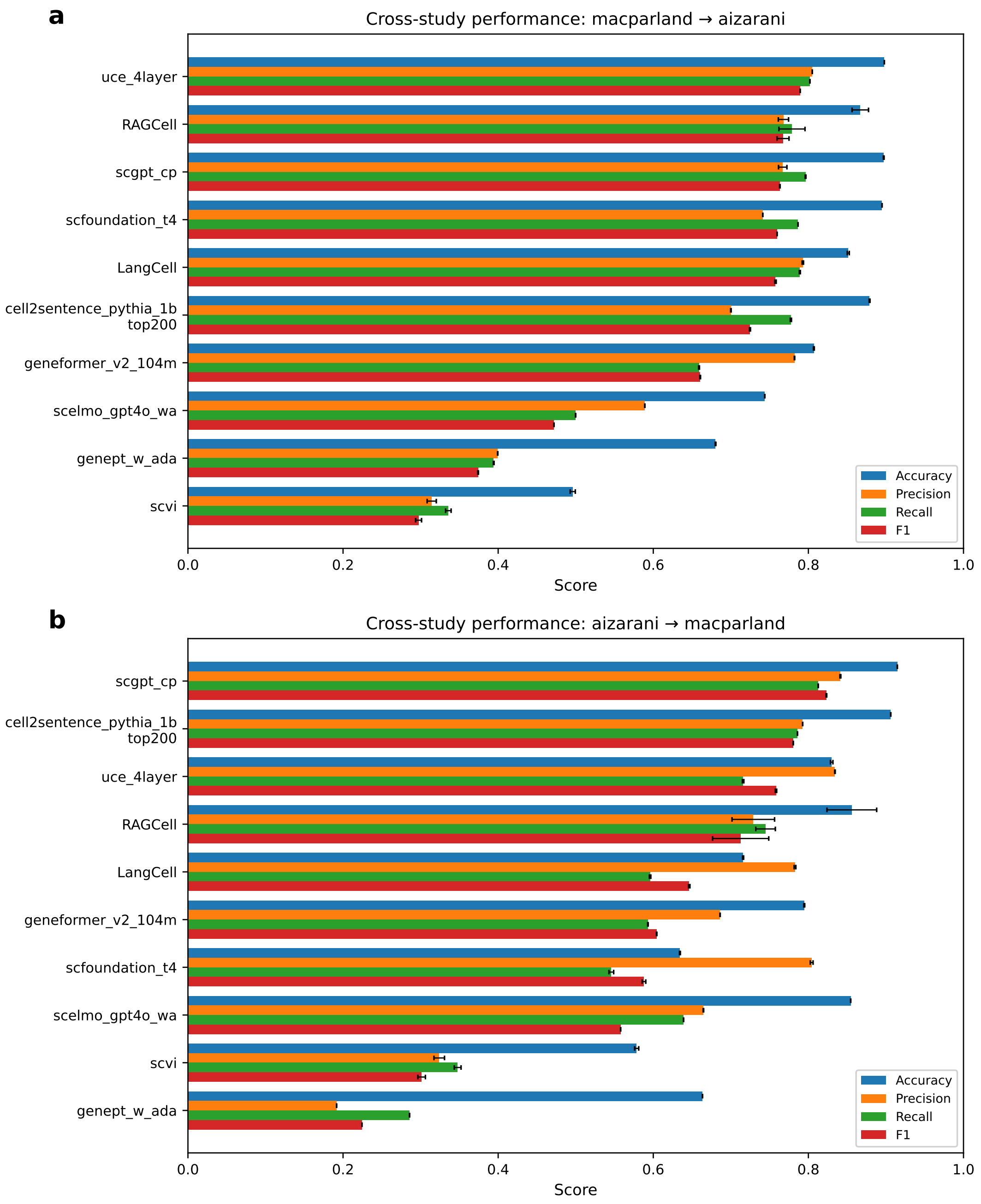

Figure S10: Benchmarking results with more methods for cell-type annotation based on the Liver dataset (cross-study comparison and 5 seeds update).

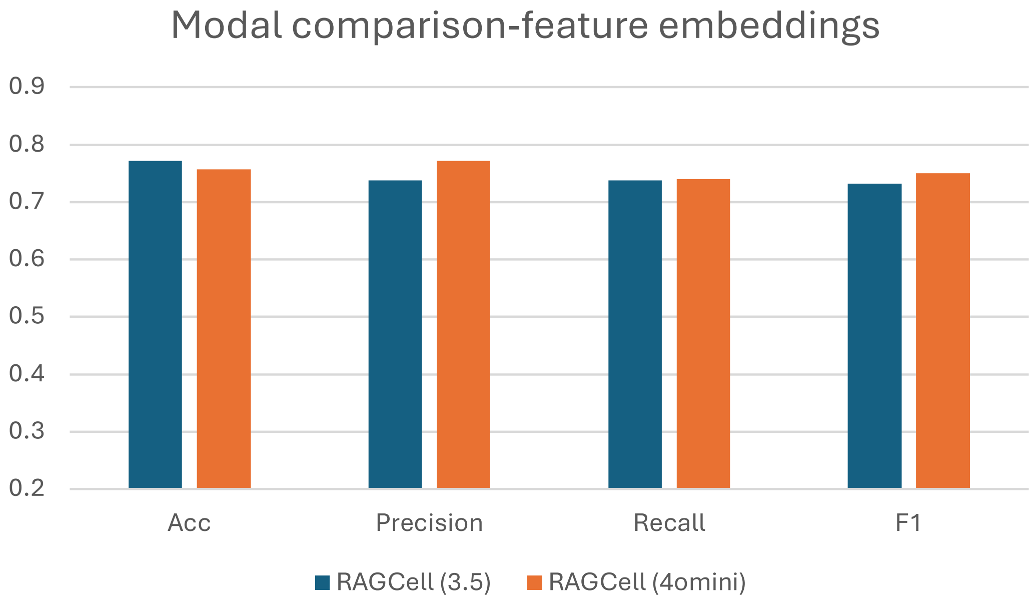

Figure S11: Ablation studies for embedding model selection.

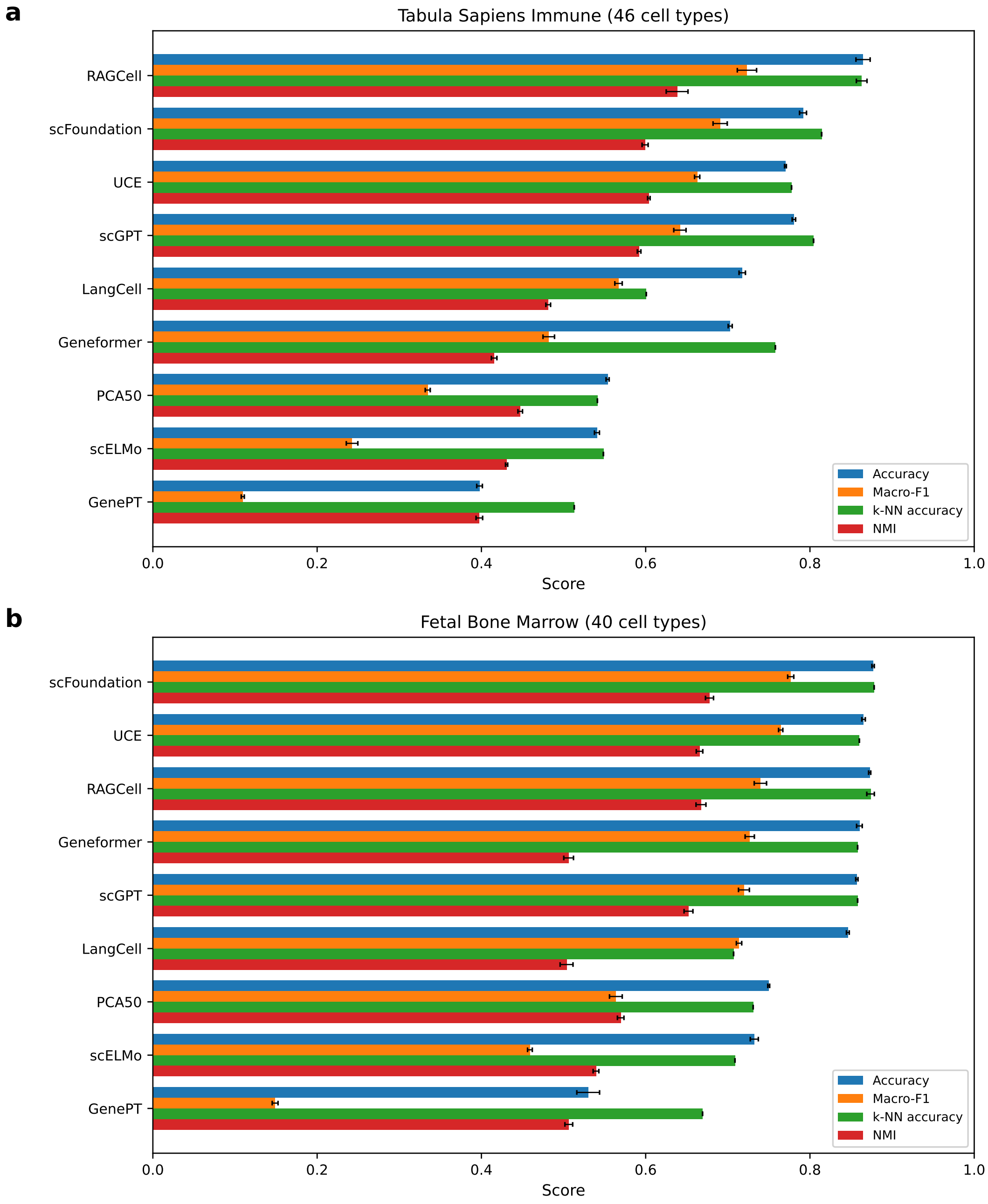

Figure S12: CTA results on two additional atlases. a, Tabula Sapiens Immune. b, Fetal Bone Marrow. Bars are means over five seeds; error bars are standard deviations.

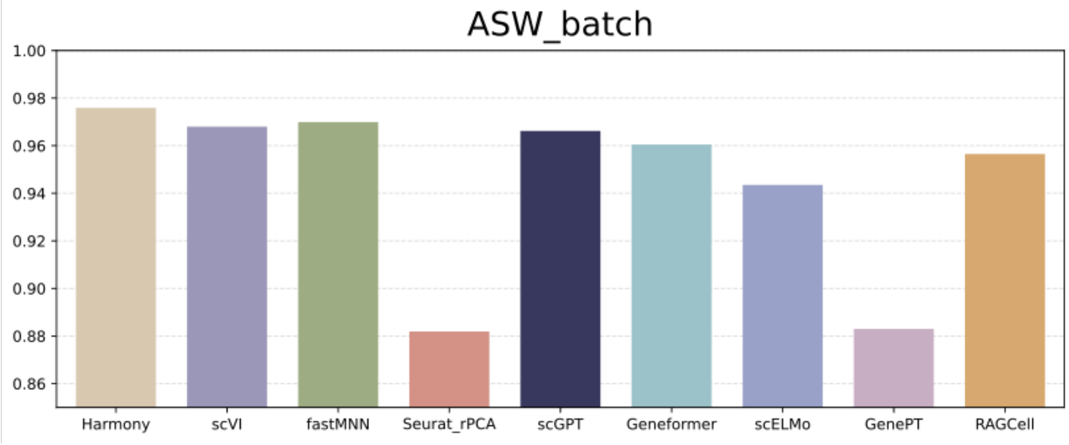

Figure S13: ASW score comparison for batch effect correction with more baselines.

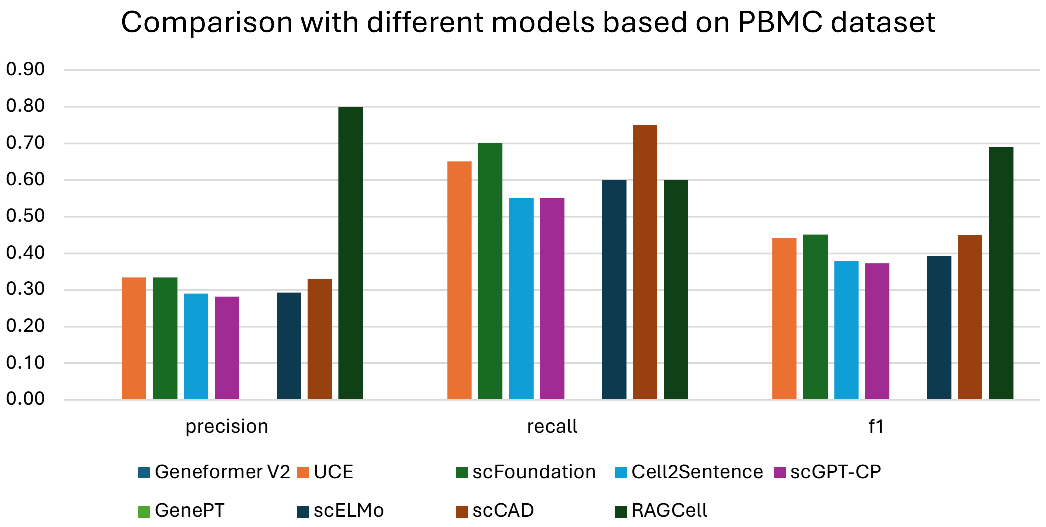

Figure S14: A more comprehensive comparison for rare cell type identification.

References

Chen, J. *et al.* (2022). Deep transfer learning of cancer drug responses by integrating bulk and single-cell rna-seq data. *Nature Communications*, **13**(1), 6494.

Chen, Y. and Zou, J. (2023). Genept: A simple but effective foundation model for genes and cells built from chatgpt. *bioRxiv*.

Cui, H. *et al.* (2024). scgpt: toward building a foundation model for single-cell multi-omics using generative ai. *Nature Methods*, pages 1–11.

Franzén, O. *et al.* (2019). Panglaodb: a web server for exploration of mouse and human single-cell rna sequencing data. *Database*, **2019**, baz046.

Gayoso, A. *et al.* (2022). A python library for probabilistic analysis of single-cell omics data. *Nature biotechnology*, **40**(2), 163–166.

Hao, M. *et al.* (2024). Large-scale foundation model on single-cell transcriptomics. *Nature Methods*, pages 1–11.

Levine, D. *et al.* (2023). Cell2sentence: Teaching large language models the language of biology. *bioRxiv*, pages 2023–09.

Liu, T. *et al.* (2023). scelmo: Embeddings from language models are good learners for single-cell data analysis. *bioRxiv*, pages 2023–12.

Rosen, Y. *et al.* (2023). Universal cell embeddings: A foundation model for cell biology. *bioRxiv*, pages 2023–11.

Satija, R. *et al.* (2015). Spatial reconstruction of single-cell gene expression data. *Nature biotechnology*, **33**(5), 495–502.

Theodoris, C. V. *et al.* (2023). Transfer learning enables predictions in network biology. *Nature*, **618**(7965), 616–624.

Vaswani, A. (2017). Attention is all you need. *Advances in Neural Information Processing Systems*.

Virshup, I. *et al.* (2023). The scverse project provides a computational ecosystem for single-cell omics data analysis. *Nature biotechnology*, **41**(5), 604–606.

Xu, Y. *et al.* (2024). sccad: Cluster decomposition-based anomaly detection for rare cell identification in single-cell expression data. *Nature Communications*, **15**(1), 7561.

Yang, F. *et al.* (2022). scbert as a large-scale pretrained deep language model for cell type annotation of single-cell rna-seq data. *Nature Machine Intelligence*, **4**(10), 852–866.

Zhao, S. *et al.* (2024). Langcell: language-cell pre-training for cell identity understanding. In *Proceedings of the 41st International Conference on Machine Learning*, ICML'24. JMLR.org.
